# Supplementary material for: Association between midday napping and long-term trajectories of cognitive function among middle-aged and older Chinese adults
Source: PLoS One. 2025 Apr 28;20(4):e0318208. doi: 10.1371/journal.pone.0318208 (PMC12036862; doi:10.1371/journal.pone.0318208)
Supplement: S1 Table — (DOCX) [file pone.0318208.s001.docx]

**S1 Table.** Description of cognitive score among the included participants (n=4648) over time from waves 1-3.

| **Characteristics** | **Wave 1** | **Wave 2** | **Wave 3** | ***P*-value** |
| --- | --- | --- | --- | --- |
| Cognitive score, mean (SD) | 15.04 (1.47) | 14.35 (2.33) | 13.95 (2.32) | <0.001 |
