# Supplementary material for: Association between midday napping and long-term trajectories of cognitive function among middle-aged and older Chinese adults
Source: PLoS One. 2025 Apr 28;20(4):e0318208. doi: 10.1371/journal.pone.0318208 (PMC12036862; doi:10.1371/journal.pone.0318208)
Supplement: S2 Table — (DOCX) [file pone.0318208.s002.docx]

**S2 Table.** Goodness-of-fit statistics of group-based trajectory analysis.

| **Number of groups** | **Trajectory shape (0=zero order, 1=linear, 2=quadratic)** | **BIC for total number of observations** | **Group proportion (%)** | **Average posterior probabilities** |
| --- | --- | --- | --- | --- |
| 2 | 0 0 | -26209.09 | 33.52/66.48 | 76.54/84.05 |
| 3 | 0 0 0 | -26167.86 | 5.57/68.76/25.67 | 75.06/76.21/72.01 |
| 4 | 0 0 0 0 | -26175.75 | 5.14/61.40/33.22/0.24 | 72.85/72.46/64.88/57.65 |
| 3 | 0 1 1 | -25441.69 | 61.68/22.76/15.56 | 77.00/81.95/74.11 |
| 3 | 1 1 1 | -25317.42 | 8.13/54.48/37.39 | 83.04/78.98/80.62 |
| 3 | 1 1 2 | -25313.66 | 8.63/55.40/35.97 | 82.48/79.38/80.16 |
| 3 | 1 2 2 | -25289.48 | 6.37/49.03/44.60 | 81.66/79.07/82.57 |
| 3 | 2 2 2 | -25191.22 | 10.22/55.74/34.04 | 83.64/80.15/80.51 |
