# Supplementary material for: Association between midday napping and long-term trajectories of cognitive function among middle-aged and older Chinese adults
Source: PLoS One. 2025 Apr 28;20(4):e0318208. doi: 10.1371/journal.pone.0318208 (PMC12036862; doi:10.1371/journal.pone.0318208)
Supplement: S3 Table — (DOCX) [file pone.0318208.s003.docx]

**S3 Table.** Sensitivity analyses of the risk of cognitive decline according to midday napping.

|  | **Case, No. (%)** | **Risk Ratio (95%CI)** | | |
| --- | --- | --- | --- | --- |
|  |  | **Model 1** | **Model 2** | **Model 3** |
| **Sensitivity 1^a^** |  |  |  |  |
| **Rapid decline** |  |  |  |  |
| Midday napping (minutes) |  |  |  |  |
| 0 | 210 (43.48) | 1.31 (0.97-1.78) | 1.40 (1.01-1.93) * | 1.39 (1.00-1.92) * |
| 1-30 | 82 (16.98) | 1 [Reference] | 1 [Reference] | 1 [Reference] |
| 31-90 | 138 (28.57) | 1.30 (0.94-1.81) | 1.43 (1.01-2.03) * | 1.44 (1.02-2.04) * |
| >90 | 53 (10.97) | 1.71 (1.12-2.62) * | 1.97 (1.26-3.08) ** | 2.00 (1.28-3.13) ** |
| **Slow decline** |  |  |  |  |
| Midday napping (minutes) |  |  |  |  |
| 0 | 1061 (41.40) | 1.20 (1.00-1.44) * | 1.22 (1.01-1.46) * | 1.22 (1.01-1.46) * |
| 1-30 | 462 (18.03) | 1 [Reference] | 1 [Reference] | 1 [Reference] |
| 31-90 | 727 (28.37) | 1.19 (0.98-1.45) | 1.25 (1.02-1.52) * | 1.25 (1.02-1.53) * |
| >90 | 313 (12.21) | 1.69 (1.30-2.18) *** | 1.81 (1.38-2.37) *** | 1.80 (1.38-2.36) *** |
| **Sensitivity 2^b^** |  |  |  |  |
| **Rapid decline** |  |  |  |  |
| Midday napping (minutes) |  |  |  |  |
| 0 | 207 (43.58) | 1.36 (1.00-1.86) * | 1.42 (1.03-1.96) * | 1.42 (1.03-1.95) * |
| 1-30 | 79 (16.63) | 1 [Reference] | 1 [Reference] | 1 [Reference] |
| 31-90 | 134 (28.21) | 1.34 (0.96-1.87) | 1.50 (1.06-2.12) * | 1.51 (1.07-2.13) * |
| >90 | 55 (11.58) | 1.85 (1.21-2.82) ** | 2.12 (1.37-3.29) ** | 2.15 (1.39-3.33) ** |
| **Slow decline** |  |  |  |  |
| Midday napping (minutes) |  |  |  |  |
| 0 | 1069 (41.26) | 1.21 (1.01-1.45) * | 1.26 (1.05-1.51) * | 1.26 (1.05-1.51) * |
| 1-30 | 467 (18.02) | 1 [Reference] | 1 [Reference] | 1 [Reference] |
| 31-90 | 739 (28.52) | 1.21 (1.00-1.47) | 1.28 (1.05-1.56) * | 1.28 (1.05-1.56) * |
| >90 | 316 (12.20) | 1.68 (1.30-2.17) *** | 1.81 (1.39-2.36) *** | 1.82 (1.40-2.37) *** |

Model 1 was adjusted for age, sex, living residence, marital status, and education level.

Model 2 was adjusted for smoking status, drinking status, social activity, depressive symptoms, functional disability, and number of chronic diseases, plus variables in model 1.

Model 3 was adjusted for nighttime sleep duration, plus variables in model 2.

^a^ After excluding participants with memory-related diseases (n=4610).

^b^ Pooled results based on 10 imputed data sets.

**P*<0.05, ***P*<0.01, ****P*<0.001.
