# Supplementary material for: Association between midday napping and long-term trajectories of cognitive function among middle-aged and older Chinese adults
Source: PLoS One. 2025 Apr 28;20(4):e0318208. doi: 10.1371/journal.pone.0318208 (PMC12036862; doi:10.1371/journal.pone.0318208)
Supplement: S4 Table — (DOCX) [file pone.0318208.s004.docx]

**S4 Table.** Risk of cognitive decline according to midday napping stratified by age and sex.

| **Subgroups** | **Midday napping (minutes)** | **Rapid decline vs. Stable** | | **Slow decline vs. Stable** | |
| --- | --- | --- | --- | --- | --- |
|  |  | **RR (95% CI)** | ***P* for interaction** | **RR (95% CI)** | ***P* for interaction** |
| **Age** |  |  | 1.000 |  | 1.000 |
| 45-59 years |  |  |  |  |  |
|  | 0 | 1.43 (0.92-2.24) |  | 1.26 (1.01-1.58) * |  |
|  | 1-30 | 1 [Reference] |  | 1 [Reference] |  |
|  | 31-90 | 1.36 (0.83-2.22) |  | 1.37 (1.08-1.75) ** |  |
|  | >90 | 1.73 (0.93-3.24) |  | 1.77 (1.28-2.43) *** |  |
| ≥60 years |  |  |  |  |  |
|  | 0 | 1.33 (0.73-2.41) |  | 0.98 (0.69-1.38) |  |
|  | 1-30 | 1 [Reference |  | 1 [Reference] |  |
|  | 31-90 | 1.78 (0.96-3.31) |  | 1.00 (0.70-1.43) |  |
|  | >90 | 2.89 (1.30-6.47) ** |  | 1.91 (1.15-3.16) * |  |
| **Sex** |  |  | 0.405 |  | 0.185 |
| Male |  |  |  |  |  |
|  | 0 | 1.18 (0.71-1.96) |  | 1.34 (1.03-1.73) * |  |
|  | 1-30 | 1 [Reference] |  | 1 [Reference] |  |
|  | 31-90 | 1.29 (0.77-2.17) |  | 1.31 (1.00-1.70) * |  |
|  | >90 | 2.22 (1.19-4.14) * |  | 1.56 (1.10-2.20) * |  |
| Female |  |  |  |  |  |
|  | 0 | 1.91 (1.13-3.24) * |  | 1.10 (0.84-1.45) |  |
|  | 1-30 | 1 [Reference] |  | 1 [Reference] |  |
|  | 31-90 | 2.06 (1.13-3.76) * |  | 1.24 (0.91-1.70) |  |
|  | >90 | 1.95 (0.84-4.54) |  | 2.31 (1.50-3.5) *** |  |

Adjusted for age, sex, living residence, marital status, education level, smoking status, drinking status, social activity, depressive symptoms, functional disability, number of chronic diseases, and nighttime sleep duration.

**P*<0.05, ***P*<0.01, ****P*<0.001.
