# Supplementary material for: Association between midday napping and long-term trajectories of cognitive function among middle-aged and older Chinese adults
Source: PLoS One. 2025 Apr 28;20(4):e0318208. doi: 10.1371/journal.pone.0318208 (PMC12036862; doi:10.1371/journal.pone.0318208)
Supplement: Attachment — Submitted filename: Response to Reviewers.docx [file pone.0318208.s006.docx]

**Manuscript title:** Association between midday napping and long-term trajectories of cognitive function among middle-aged and older Chinese adults

**Submission ID:** PONE-D-24-03278

**Authors:** Jinghong Huang; Dongrui Peng; Xiaohui Wang

**Journal:** *PLOS ONE*

**Type:** Research Paper

**Submitted on:** Jan 25 2024

Dear Editor,

We gratefully thank you and all reviewers for the constructive remarks and useful suggestions, which have significantly enabled us to improve the manuscript. We are delighted at your decision to invite us to revise and resubmit this manuscript.

The manuscript has been revised according to the comments from the editor and reviewers, and all revisions have been used tracked changes for ready identification. The Point-by-point response to reviewers can be found below. We also prepared the track change and unmarked version for your reference and shared it with editor and reviewers.

We hope this revision is satisfactory and our manuscript will be acceptable for publication in the journal.

Sincerely,

Authors

**Point-by-point response to editor and reviewers**

**Editor**

***COMMENTS***

1. When submitting your revision, we need you to address these additional requirements.

Please ensure that your manuscript meets PLOS ONE's style requirements, including those for file naming. The PLOS ONE style templates can be found at

https://journals.plos.org/plosone/s/file?id=wjVg/PLOSOne_formatting_sample_main_body.pdf and

https://journals.plos.org/plosone/s/file?id=ba62/PLOSOne_formatting_sample_title_authors_affiliations.pdf.

**Response:** Thank you for your comment.

We have carried out a careful check and have met the above requirements.

2. We note that the grant information you provided in the ‘Funding Information’ and ‘Financial Disclosure’ sections do not match.

When you resubmit, please ensure that you provide the correct grant numbers for the awards you received for your study in the ‘Funding Information’ section.

**Response:** We gratefully appreciate your careful check.

When we submitted our revisions, we did not see the "Financial Disclosure" sections in the submission system. Therefore, we are stating "Financial Disclosure Statement" here: This study was supported by the National Natural Science Foundation of China (Grant No. 72274088). The funders had no role in study design, data collection and analysis, decision to publish, or preparation of the manuscript.

3. We note that you have indicated that there are restrictions to data sharing for this study. PLOS only allows data to be available upon request if there are legal or ethical restrictions on sharing data publicly. For more information on unacceptable data access restrictions, please see <http://journals.plos.org/plosone/s/data-availability#loc-unacceptable-data-access-restrictions>.

Before we proceed with your manuscript, please address the following prompts:

a) If there are ethical or legal restrictions on sharing a de-identified data set, please explain them in detail (e.g., data contain potentially identifying or sensitive patient information, data are owned by a third-party organization, etc.) and who has imposed them (e.g., a Research Ethics Committee or Institutional Review Board, etc.). Please also provide contact information for a data access committee, ethics committee, or other institutional body to which data requests may be sent.

b) If there are no restrictions, please upload the minimal anonymized data set necessary to replicate your study findings to a stable, public repository and provide us with the relevant URLs, DOIs, or accession numbers. For a list of recommended repositories, please see

https://journals.plos.org/plosone/s/recommended-repositories. You also have the option of uploading the data as Supporting Information files, but we would recommend depositing data directly to a data repository if possible.

We will update your Data Availability statement on your behalf to reflect the information you provide.

**Response:** We gratefully appreciate your careful check.

There are no restrictions.

Data are from the China Health and Retirement Longitudinal Study(CHARLS) and are publicly available from the CHARLS website: <https://charls.charlsdata.com/pages/data/111/zh-cn.html>. Dataset titles used in this study were 2015 CHARLS Wave 3, 2013 CHARLS Wave2, and 2011 CHARLS Wave1). The present study's authors had no special privileges in accessing these datasets which other interested researchers would not have.

Our URL has no fault, which is consistent with the previous CHARLS study published in PLOS ONE. DOI: 10.1371/journal.pone.0245670; DOI: 10.1371/journal.pone.0226279.

**Reviewer 1**

***COMMENTS***

1. the authors need to address on the full time/duration of the night sleep, at least as the covariates in logistical regression. Also, the sleep quality and pattern need to be addressed and discussed. So, it had better re-do the data analysis first.

**Response:** We gratefully appreciate your constructive suggestion.

Thank you for your constructive comments. Firstly, the nighttime sleep duration was included as a covariate in logistic regression model. However, we missed that variable in the analysis of “Association of change in midday napping over 4-years with cognitive decline risk”. Therefore, for this part of the results, we re-analysed the results after also incorporating nighttime sleep duration as a covariate. *See Table 3*, which is generally consistent with the previous results.

We similarly considered that midday napping, nighttime sleep duration, and sleep quality are closely related to each other due to circadian rhythms. Therefore, we included nighttime sleep duration and sleep quality-related variables at baseline, in order to examine longitudinal association between midday napping and trajectories of cognitive function. However, the present study had adjusted for depression as a covariate, rather than sleep quality, because:

1. In the CHARLS database, sleep quality is included as part of the assessment of depression (the CESD-10 scale is used to assess depression). 10-items of CESD-10 scale includes: (1) bothered by little things, (2) had trouble concentrating, (3) felt depressed, (4) everything was an effort, (5) felt hopeless, (6) felt fearful, (7) sleep was restless, (8) felt unhappy, (9) felt lonely, and (10) could not get going. Each item was scored as follows: 0 (rarely or none of the time, <1 day), 1 (some or little of the time, 1–2 days), 2 (occasionally or a moderate amount of the time, 3–4 days), and 3 (most or all of the time, 5–7 days). The item “sleep was restless” is always used to assess sleep quality in CHARLS research[1, 2].

2. Depression, as a common psychological factor, may have an impact on the relationship between midday napping and cognitive function. The main points are: (1) Depression may affect cognitive function: patients with depression are often accompanied by impairment of cognitive function, such as diminished attention, memory, and executive function. (2) Depression may affect napping habits: depressed patients may have sleep disorders, such as difficulty in falling asleep and waking up early, and these symptoms may affect patients' midday napping habits. Therefore, adjusting for depression as a covariate can reduce the interference of depression (including the assessment of sleep quality) on the results of the study, thus increasing the sensitivity of the study of the relationship between midday napping and cognitive function, making the results more accurate and reliable.

In summary, we have made improvement in response to your valuable comments:

In the Discussion section, we have added a discussion on sleep quality. The revised text states: *For example, a clinical study found that Tau proteins damage brain regions that regulate wakefulness and sleep states, leading to an increase in napping, which affects circadian rhythms and leads to disrupted day and night sleep rhythms and worsen sleep quality, which in turn leads to more Tau proteins and amyloid-like proteins clumping together in the brain, further deteriorating cognitive function[3]. Moreover, combined with the concrete context of education and social development in middle-aged and older Chinese adults. First, they may easily experience sleep deprivation due to work and deterioration of social functions, respectively[4, 5], which is consistent with a study based on a Chinese population. The study found that the relationship between sleep duration and sleep quality varied by occupation, e.g. blue-collar workers were at higher risk of experiencing shorter sleep duration and poorer sleep quality, which may be due to differences in socio-economic status[6]. And these sleep disorder may affect sleep recovery beyond two days, further affecting cognitive function[7]. There also seems to be a suggestion that subsequent research could explore the relationship between sleep and cognitive function from the perspective of different occupations. (Pages 16-17, Lines 203-211)*

**Reviewer 2**

***COMMENTS***

1. This study was conducted very well with large number of participants and statistic analysis was nicely performed.

However, the introduction was not declaring the title enough about the midday napping itself.

**Response:** Thanks for your professional review of our study.

We added a statement about midday napping to better respond to the title and bridge the context. The added text state: *Midday napping is considered a healthy lifestyle with intervening properties. The prevalence of habitual midday napping ranges from 22% to 69% among older adults worldwide[8-11]. Meanwhile, midday napping is also prevalent in China, as Chinese culture considers it an important part of healthy living[10]. (Page 3, Lines 38-40)*

2. Authors highlighted a rapid and slow cognitive decline, which was very interesting, nevertheless it was not mention at all in the introduction nor methods and how it was calculated statistically. The figure 2 could be more statistically informative. It was also interesting, how the rapid cognitive decline was only found until 2013, was there any explanation?

**Response:** Thank you for your suggestion.

In the introduction section, we add research on trajectories of cognitive function. The added text states: *However, these studies assessed cognitive function at a single point in time. This approach does not capture the dynamic process of cognitive function, which may be important because middle-aged and older adults appear to experience different patterns of cognitive function over time. For example, in a study of body mass index and trajectories of cognitive function in 5693 Chinese middle-aged and older adults (aged 45 years+), it was found that individuals tended to show the following three longitudinal patterns of cognitive function, including rapid decline, slow decline, and stable[12]. (Page 4, Lines 50-54)*

We had a description of the trajectory methodology and statistical calculations in the Methods section *(Page 8, Lines 111-116)*. In addition, in Supplementary file “S2 Table”, we had statistics related to trajectory determination.

We have added more statistical information to Figure 2, namely the proportion of samples in each trajectory group, which will be consistent with the presentation of previous trajectory-related studies[12]. We believe this will be more helpful to the reader. *See Figure 2*.

Similar trajectories were also found in a cohort study of body mass index and trajectories of cognitive function[12]. In this study, the rapid decline in cognitive function that occurred in 2011-2013 may have been due to the fact that participants in the rapid decline group were likely to be older, rural residence, not married/cohabitated, lower education level (no former education and primary school), current smoker, no social activity, depressed, severe functional disability, and shorter nighttime sleep duration than slow decline and stable groups. These factors are often considered risk factors for cognitive impairment.

3. In the discussion authors mention about unhealthy midday napping, and it only appeared once without prior introduction.

**Response:** Thank you for your comment.

Our have added the information about unhealthy midday napping. The revised text states: *Participants who* *persistently had unhealthy midday napping (transferred from >90 to 31-90 minutes, switched from 31-90 to >90 minutes, and persisted in >90 minutes) were at increased risk for trajectories of cognitive decline, especially rapid decline. (Page 15, Lines 179-181)*

**Reference**

1.	Li M, Wang N, Dupre ME. Association between the self-reported duration and quality of sleep and cognitive function among middle-aged and older adults in China. Journal of Affective Disorders. 2022;304:20-7. doi: 10.1016/j.jad.2022.02.039. PubMed PMID: WOS:000764263400003.

2.	Zhao Y, Hu Y, Smith JP, Strauss J, Yang G. Cohort Profile: The China Health and Retirement Longitudinal Study (CHARLS). International Journal of Epidemiology. 2014;43(1):61-8. doi: 10.1093/ije/dys203. PubMed PMID: WOS:000332341300012.

3.	Oh J, Eser RA, Ehrenberg AJ, Morales D, Petersen C, Kudlacek J, et al. Profound degeneration of wake-promoting neurons in Alzheimer's disease. Alzheimers & Dementia. 2019;15(10):1253-63. doi: 10.1016/j.jalz.2019.06.3916. PubMed PMID: WOS:000490148500002.

4.	Hendriks S, Peetoom K, Bakker C, van der Flier WM, Papma JM, Koopmans R, et al. Global Prevalence of Young-Onset Dementia A Systematic Review and Meta-analysis. Jama Neurology. 2021;78(9):1080-90. doi: 10.1001/jamaneurol.2021.2161. PubMed PMID: WOS:000676017800004.

5.	Lee Y, Chi I, Palinkas LA. Retirement, Leisure Activity Engagement, and Cognition Among Older Adults in the United States. Journal of Aging and Health. 2019;31(7):1212-34. doi: 10.1177/0898264318767030. PubMed PMID: WOS:000474901300009.

6.	Sun W, Yu Y, Yuan J, Li C, Liu T, Lin D, et al. Sleep Duration and Quality among Different Occupations-China National Study. Plos One. 2015;10(3). doi: 10.1371/journal.pone.0117700. PubMed PMID: WOS:000351284600015.

7.	Laharnar N, Fatek J, Zemann M, Glos M, Lederer K, Suvorov AV, et al. A sleep intervention study comparing effects of sleep restriction and fragmentation on sleep and vigilance and the need for recovery. Physiology & Behavior. 2020;215. doi: 10.1016/j.physbeh.2019.112794. PubMed PMID: WOS:000512221600022.

8.	dos Santos AA, Ceolim MF, Iost Pavarini SC, Neri AL, Rampazo MK. Association between sleep disorders and frailty status among elderly. Acta Paulista De Enfermagem. 2014;27(2):120-5. doi: 10.1590/1982-0194201400022. PubMed PMID: WOS:000338330900006.

9.	Fang W, Li Z, Wu L, Cao Z, Liang Y, Yang H, et al. Longer habitual afternoon napping is associated with a higher risk for impaired fasting plasma glucose and diabetes mellitus in older adults: results from the Dongfeng-Tongji cohort of retired workers. Sleep Medicine. 2013;14(10):950-4. doi: 10.1016/j.sleep.2013.04.015. PubMed PMID: WOS:000324103600005.

10.	Li J, Cacchione PZ, Hodgson N, Riegel B, Keenan BT, Scharf MT, et al. Afternoon Napping and Cognition in Chinese Older Adults: Findings from the China Health and Retirement Longitudinal Study Baseline Assessment. Journal of the American Geriatrics Society. 2017;65(2):373-80. doi: 10.1111/jgs.14368. PubMed PMID: WOS:000397006700024.

11.	Pace-Schott EF, Spencer RMC. Age-related changes in the cognitive function of sleep. In: Green AM, Chapman CE, Kalaska JF, Lepore F, editors. Enhancing Performance for Action and Perception: Multisensory Integration, Neuroplasticity and Neuroprosthetics, Pt I. Progress in Brain Research. 1912011. p. 75-89.

12.	Zhang W, Chen Y, Chen N. Body mass index and trajectories of the cognition among Chinese middle and old-aged adults. Bmc Geriatrics. 2022;22(1). doi: 10.1186/s12877-022-03301-2. PubMed PMID: WOS:000829153500003.
