# Supplementary material for: Association between midday napping and long-term trajectories of cognitive function among middle-aged and older Chinese adults
Source: PLoS One. 2025 Apr 28;20(4):e0318208. doi: 10.1371/journal.pone.0318208 (PMC12036862; doi:10.1371/journal.pone.0318208)
Supplement: Attachment — Submitted filename: Response_to_Reviewers_auresp_3.docx [file pone.0318208.s008.docx]

**Manuscript title:** Association between midday napping and long-term trajectories of cognitive function among middle-aged and older Chinese adults

**Submission ID:** PONE-D-24-03278R2

**Authors:** Jinghong Huang; Dongrui Peng; Xiaohui Wang

**Journal:** *PLOS ONE*

**Type:** Research Paper

**Submitted on:** Jan 25 2024

Dear Editor,

We gratefully thank you and all reviewers for the constructive remarks and useful suggestions, which have significantly enabled us to improve the manuscript. We are delighted at your decision to invite us to revise and resubmit this manuscript.

The manuscript has been revised according to the comments from the editor and reviewers, and all revisions have been used tracked changes for ready identification. The Point-by-point response to reviewers can be found below. We also prepared the track change and unmarked version for your reference and shared it with editor and reviewers.

We hope this revision is satisfactory and our manuscript will be acceptable for publication in the journal.

Sincerely,

Authors

**Point-by-point response to reviewers**

**Reviewer 3**

***COMMENTS***

1. I commend the authors for their thorough revisions and thoughtful responses to the reviewers' comments. The manuscript is significantly improved, and the revisions strengthen its scientific rigor.

Data Reanalysis and Statistical Rigor: The reanalysis of the data, with additional covariates included, is well-executed. The use of multinomial logistic regression is well-justified, and the sample size balance across trajectory groups enhances the robustness of the findings.

Biological Mechanism Discussion: The expanded discussion of biological mechanisms, including Tau protein accumulation and inflammation, strengthens the manuscript and provides a solid biological basis for the observed relationships.

Literature Review: The authors have improved the introduction by including relevant studies from the CHARLS database, providing important context for their findings and justifying their methodology.

Mental Health Factors: The inclusion of sleep quality as an independent variable adds clarity to the analysis and strengthens the validity of the conclusions.

**Response:** We are pleased to know that the revised manuscript has improved in scientific rigor and overall quality. Thank you very much for your positive feedback.

**Reviewer 4**

***COMMENTS***

1. The study initially targeted a group of over 17705 people, which at first glance appears to be a large sample. However, the actual number of participants that remained in the final analysis was only around 4648, which is merely about 25% of the original group. The almost majority of the original participants cannot be used for this study, indicating the potential for significant bias. Despite this, there is no mention of any discussion or acknowledgment of this limitation in the text.

**Response:** Thank you for your insightful comment.

We acknowledge that this substantial attrition (approximately 25% of the original sample) represents a limitation of the study and may have introduced selection bias, potentially affecting the generalizability of the findings. To address this, we have revised the "Limitations" section of the manuscript to explicitly acknowledge this issue and its potential implications. Specifically, we have added the following text: *Finally, the sample size was significantly reduced from the initial 17705 participants to the final 4648 (approximately 25% of the original sample) due to the application of exclusion criteria. While these criteria were designed to ensure data quality and homogeneity, this substantial attrition may have introduced selection bias, potentially affecting the generalizability of the findings. Future research should consider the above limitations. (Page 20, Lines 704-707)*

2. MMSE and TICS-10 are two tools to assess cognitive function. However in this work, the authors stated this study used MMSE, and then used TICS-10. Readers will be confused by these.

**Response:** We gratefully appreciate your constructive suggestion. To ensure clarity and avoid confusion, we have revised the manuscript accordingly. Specifically, the text now reads: *The dependent variable in this study was long-term trajectories of cognitive function. Cognitive function represented the* three ability assessments: orientation, attention, episodic memory, and visual-spatial, *measured at baseline and at 2 subsequent follow-up visits (2013 and 2015).* *The cognitive function measures in CHARLS were derived from the components of the Telephone Interview for Cognitive Status battery (TICS-10), which specifically assessed orientation and attention[1]. (Pages 6-7, Lines 90-101)*

We believe this revised description accurately reflects the methodology and eliminates any ambiguity for the readers. Thank you for pointing out this important detail.

3. Despite the large sample size, it is recommended to perform and report in the text a normality test of the distribution (example: Shapiro-Wilk).

**Response:** We appreciate your attention to the robustness of the statistical analysis.

Among the covariates included in this study, only age and nighttime sleep duration are continuous variables. In response to your suggestion, we conducted Shapiro-Wilk tests for these two variables. The results indicated that both age and nighttime sleep duration did not strictly follow a normal distribution (*P*<0.05). However, the primary statistical method employed in this study—multinomial logistic regression—does not assume normality of the covariates. Therefore, this deviation from normality does not affect the validity of our findings.

To clarify this, we have added the following statement to the Methods section of the revised manuscript: *The normality of continuous variables (age and nighttime sleep duration) was assessed using the Shapiro-Wilk test. The results showed that these variables did not follow a normal distribution (P<0.05). However, as multinomial logistic regression does not require covariates to be normally distributed, this deviation does not affect the validity of the analysis. (Page 9, Lines 156-158)*

We hope this revision adequately addresses your concern and enhances the methodological clarity of our manuscript. Thank you again for your valuable feedback.

4. In the introduction, the authors provide information about the association between sleep and cognitive function. In my opinion, one aspect you could introduce into the introduction of you paper is the relationship between sleep, physical capability, and cognitive function. The article (PMID: 37084959; 38835194) may be helpful in this section. In this way, the introduction will be more comprehensive.

**Response:** We thank your insightful suggestion.

While the primary focus of our study is the association between midday napping and long-term cognitive function trajectories, we agree that considering the role of physical capability could provide a broader perspective on the factors influencing cognitive health. To address this, we have incorporated a forward-looking discussion in the manuscript to highlight this point: *It is worthwhile that cognitive function is also influenced by other factors such as physical capability (including handgrip strength and walking speed)[2, 3]. Importantly, there may be a bidirectional relationship between them and midday napping, e.g., improved physical capability may enhance the restorative effects of napping, while adequate napping may promote physical recovery by restoring energy and reducing fatigue. This interaction between physical ability and midday napping may have a cumulative effect on cognitive health. Therefore, future research is needed to explore the association between the interaction of napping and physical ability and cognitive function. (Pages 19, Lines 692-696)*

This addition has been placed in the discussion section to ensure the flow of the introduction remains focused on the study objectives. By incorporating this perspective, we aim to underscore the complexity of factors influencing cognitive function and provide directions for future research. We sincerely thank the reviewer for this valuable suggestion, which has enriched the scope of our discussion.

5. It is important to determine the relationship according to sex and age group, since sex and age difference (e.g.: middle-aged, young-old, and old-old) may be existed in the relationship.

**Response:** Thank you for highlighting the importance of examining potential differences in the relationship between midday napping and cognitive function trajectories across age and sex groups.

In response to your suggestion, we conducted stratified analyses and interaction tests by both age (45-59 and ≥60 years) and sex (male and female). As shown in *S4 Table*, the results indicate that the differences in the relationship between midday napping and long-term trajectories of cognitive function across these subgroups are not statistically significant (all *P* value for interaction>0.05).

To ensure clarity and rigor, we have described the stratified analyses and interaction testing in detail in the Methods section *(Page 9, Lines 162-164)*, and the findings have been incorporated into the Supporting Information. These results suggest that while there may be variability in the observed associations, the differences by age and sex are not statistically significant.

Thanks again for your insightful suggestion, which has allowed us to more comprehensively address potential modifiers of the relationship in our study.

**Reviewer 5**

***COMMENTS***

1. I’m not sure why the authors are masking the impact of emotional or psychiatric problems by lumping them in with chronic physical diseases. This should be a separate variable, particularly as it can affect falling to sleep and sleep quality, and cognitive function, regardless of a person’s physical health problems. (Brown & Mutambudzi, 2023, Aging & Mental Health, 27(6), 1095–1102). Furthermore, mental health factors – emotional and psychiatric problems and CES-D depressive symptoms – have a separate relationship to cognitive function, and should therefore be considered as separate measures in the models. However, the authors are not including CES-D scores in their models, even though their response to the previous review implies that they had initially included this measure. I think excluding depression is a mistake, because it definitely impacts sleep.

**Response:** Thank you for your valuable comments.

We have made some clarifications and adjustments regarding the issue of grouping emotional or psychiatric problems with chronic physical diseases.

In the CHARLS database, emotional or psychiatric problems are included alongside 13 other chronic diseases (such as hypertension, diabetes, etc.) as self-reported conditions. To maintain consistency with the database, we initially listed them together as covariates in our model. However, we understand your concern that emotional or psychiatric problems may independently impact outcomes such as sleep quality and cognitive function, and thus combining them with other chronic diseases may mask their unique effects.

In response, we attempt to treat emotional or psychiatric problems as a separate variable. Specifically, individuals with emotional or psychiatric problems are classified as having depressive symptoms. This approach allows us to account for the independent effects of emotional disorders on the outcomes and ensures their analysis. Consequently, we have removed emotional or psychiatric problems from the list of number of chronic diseases in the covariates to avoid duplication.

We will update the methods section as follows: *Physician-diagnosed comorbidities were self-reported and included hypertension, dyslipidemia, diabetes, cancer, chronic lung disease, liver disease, heart problems, stroke, kidney disease, digestive disease, memory-related disease, arthritis or rheumatism, and asthma. (Page 8, Lines128-129)*

Summarily, in the updated analysis, emotional or psychiatric problems, together with depressive symptoms measured using the CESD-10 scale were treated as a separate variable and were not included in the number of chronic diseases to avoid duplication. Specifically, the text now reads: *Depressive symptoms were measured using the 10-item Center for Epidemiological Studies Depression Scale (CESD-10), and scores greater than or equal to 12 were considered having depressive symptoms, otherwise not[4]. Additionally, individuals with physician-diagnosed emotional or psychiatric problems were also considered to have depressive symptoms. (Page 8, Lines 123-125)*

Moreover, we would like to provide clarification regarding our approach to mental health factors and sleep quality in the models.

We acknowledge your point that mental health factors, such as depressive symptoms (CESD-10), should be considered as separate measures in the models. In response, we have carefully reconsidered the role of psychological factors in our analysis and have decided to revert and update to our original approach, where depressive symptoms will be included as a covariate in our model. This decision was driven by several key considerations:

1. Consistent and Updated with Previous Analysis: In our initial analysis, we considered depressive symptoms measured using the CESD-10 scale as a separate covariate because the CESD-10 scale includes a sleep quality-related item (DC015: "My sleep was restless"). Including depressive symptoms as a covariate allows us to better capture the broader impact of psychological well-being on cognitive functioning while avoiding redundancy between the sleep quality item and depressive symptoms. Meanwhile, to comprehensively measure mental health factors, emotional or psychiatric problems, together with depressive symptoms measured using the CESD-10 scale were treated as a separate variable.
2. Avoiding Redundancy and Overlap: The sleep quality question (DC015) is part of the CESD-10 scale, which makes it inherently linked to depressive symptoms. Given this overlap, we felt that keeping the sleep quality item (DC015) in the model alongside depressive symptoms would risk introducing multicollinearity. Hence, we decided to use depressive symptoms as a comprehensive measure to reflect both sleep quality and depressive symptoms in a unified way.
3. Addressing Pre-Reviewer Feedback: While the suggestion to consider mental health factors as confounders rather than mediators is valuable, we consider that depressive symptoms serve as a well-established and comprehensive measure of psychological distress, which justifies its inclusion in the model. This inclusion is consistent with prior research that has linked depressive symptoms to cognitive functioning and underscores the importance of mental health in our analysis.

We are aware that this adjustment may appear as a shift in our analysis strategy. However, we would like to emphasize that our approach is rooted in the goal of providing the most scientifically rigorous and accurate model. After thoroughly considering your comment on mental health as a covariate, we believe that reverting to our original analysis is the most methodologically sound decision. We hope that this rationale clarifies our decision and we welcome any further suggestions from the reviewer.

*In our updated analysis, therefore, the independent variable (X=midday napping) remains unchanged, while the dependent variable continues to be trajectories of cognitive function. The following covariates were included in the model: age, sex, living residence, marital status, education level, smoking status, drinking status, social activity, depressive symptoms, functional disability, number of chronic diseases, and nighttime sleep duration. Age and nighttime sleep duration were treated as continuous variables, while the others were categorical.*

Thanks again for your suggestion, which has helped us further refine our analysis.

2. The authors do not clearly state they type of variable (dichotomous/nominal, ordinal, continuous/interval) for each covariate they are defining on pages 7-8. For example, they never defined their age variable – is it continuous or are they looking at age groups?

**Response:** We sincerely appreciate your valuable comment.

We apologize for any confusion regarding the definition of the variables. To clarify, the types of variables included in our models are as follows:

Age and nighttime sleep duration were treated as continuous variables.

All other covariates (including sex, living residence, marital status, education level, smoking status, drinking status, social activity, depressive symptoms, functional disability, and number of chronic diseases) were treated as categorical variables.

Specifically, the text now reads: *Age and nighttime sleep duration were treated as continuous variables, while the others (including sex, living residence, marital status, education level, smoking status, drinking status, social activity, depressive symptoms, functional disability, and number of chronic diseases) were categorical. (Page 7, Lines 111-113)*

3. How are they dealing with missingness in these variables?

**Response:** Thanks for your constructive suggestion.

In our main analysis, we did not exclude cases with missing covariates. However, we utilized multiple imputation to address missingness in the covariates during our sensitivity analysis. Specifically, we used the multiple imputation by chained equations (MICE) method to create 10 imputed datasets. The results from these multiple imputations were then pooled using the "mi estimate" command in Stata software to account for uncertainty in the imputation process. *See Page 10, Lines 169-170.*

The results obtained from the imputed datasets were consistent with those from the primary analysis, indicating that the handling of missing data did not substantially affect our findings. This approach is in line with best practices for dealing with missing data and ensures the robustness of our results. *See S3 Table.*

We hope this sufficiently addresses your concern.

4. Drinking status – what are they doing with people who never drink? Aren’t they substantively different from those who drink occasionally? And on what basis have they chosen to define “not occasional drinking” as more than or equal to 4-6 times a week? Is that just the way the categories are defined in the survey?

**Response:** Thank you for your insightful questions regarding drinking status.

We agree that never drinkers may differ substantively from those who drink occasionally. However, in our initial analysis, we followed the CHARLS database categorization of alcohol consumption [including (1) Once a month, (2) 2-3 times a month, (3) Once a week, (4) 2-3 times a week, (5) 4-6 times a week, (6) Once a day, (7) Twice a day, and (8) More than twice a day], which did not explicitly differentiate nondrinkers from occasional drinkers. To address this, we have reclassified the definition of drinking status, explicitly categorizing drinking status into nondrinker, light to moderate drinker (<twice a day currently), and heavy drinker (≥twice a day currently). This refinement ensures that the unique characteristics of nondrinkers are appropriately considered in our analysis. These adjustments ensure that the categorization is both consistent with the CHARLS definitions and aligned with established literature[5] while capturing a clear separation between different drinking behaviours.

Meanwhile, the definition of smoking status was also similarly adjusted. Smoking status was defined as nonsmoker, light to moderate (<20 cigarettes/day currently or history of smoking), and heavy (≥20 cigarettes/day currently)[5].

We have updated the Methods section (*See Pages 7-8, Lines 115-121)* to reflect these changes and have reanalyzed our models accordingly. The results remain robust, and no substantial differences were observed in the primary associations of interest when using the revised categorizations.

Thanks again for your constructive feedback, which has allowed us to refine our methodology and improve the clarity of our analysis.

5. Please state clearly which variable is your dependent variable – is it midday napping, or is it cognitive function? When you introduce your measures (line 83), you are not labeling these 2 measures as either dependent or independent variables in your models. Don’t make the reader wait until your statistical analyses.

**Response:** Thank you for your thoughtful comment.

We appreciate your suggestion to clearly label the dependent and independent variables when introducing the measures. In response, we have updated the Methods section to clearly state the roles of our key variables. Specifically, we have clarified that:

*The independent variable in this study was baseline (2011) midday napping, which was self-reported. (Page 6, Line 86)*

*The dependent variable in this study was long-term trajectories of cognitive function. (Page 6, Line 90)*

**Reference**

1.	Liu Y, Liu Z, Liang R, Luo Y. The association between community-level socioeconomic status and cognitive function among Chinese middle-aged and older adults: a study based on the China Health and Retirement Longitudinal Study (CHARLS). Bmc Geriatrics. 2022;22(1). doi: 10.1186/s12877-022-02946-3. PubMed PMID: WOS:000772066000001.

2.	Zhao X, Chen S, Liu N, Hu F, Yu J. Handgrip strength is positively associated with successful aging in older adults: A national cross-sectional study in China. Journal of Affective Disorders. 2023;333:30-7. doi: 10.1016/j.jad.2023.04.041. PubMed PMID: WOS:000987093000001.

3.	Zhao X, Zhang H, Yu J, Liu N. Independent and combined associations of handgrip strength and walking speed with cognitive function in older adults: evidence from a national cross-sectional study. Aging & Mental Health. 2024;28(12):1659-66. doi: 10.1080/13607863.2024.2360018. PubMed PMID: WOS:001242450400001.

4.	Chen H, Mui AC. Factorial validity of the Center for Epidemiologic Studies Depression Scale short form in older population in China. International Psychogeriatrics. 2014;26(1):49-57. doi: 10.1017/s1041610213001701. PubMed PMID: WOS:000332513600007.

5.	Li J, Cacchione PZ, Hodgson N, Riegel B, Keenan BT, Scharf MT, et al. Afternoon Napping and Cognition in Chinese Older Adults: Findings from the China Health and Retirement Longitudinal Study Baseline Assessment. Journal of the American Geriatrics Society. 2017;65(2):373-80. doi: 10.1111/jgs.14368. PubMed PMID: WOS:000397006700024.
